# Workshop report: The role of Earth Observation for multi-(hazard-)risk assessment and management

**DOI:** 10.1016/j.isci.2024.110833

**Published:** 2024-10-14

**Authors:** Nicole van Maanen, Marleen de Ruiter, Philip J. Ward

**Affiliations:** 1 Institute for Environmental Studies (IVM), Vrije Universiteit Amsterdam, Amsterdam, the Netherlands

## Abstract

At the 2024 General Assembly of the European Geophysical Union, a workshop was organized to introduce the EO4Multihazards project and its three main research questions. The workshop included a project overview, panel discussion, group activities, and a feedback session. It also showcased an interactive poster. These efforts enriched our discussions and broadened our understanding of Earth Observation’s role in multi-hazard risk assessment and management. This document summarizes these discussions.

**Figure:**
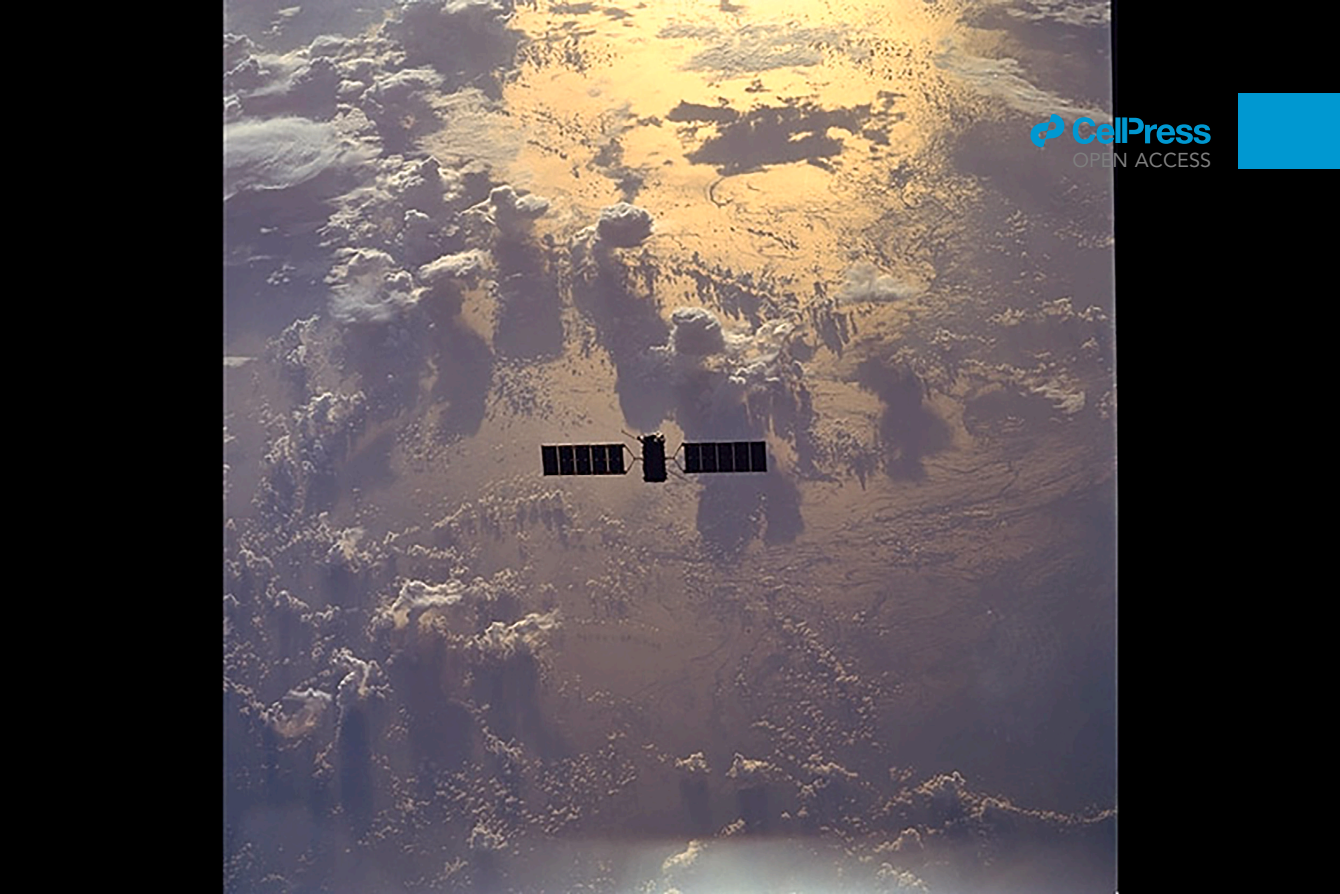
Above image: Eureca deployment. Photo: ©ESA.

Understanding the role of EO is vital for improving multi-(hazard-) risk assessment and enhancing community resilience

Integrating social-related indicators and transitioning physical data into social proxies deepens our comprehension of vulnerability dynamics and societal impacts.

## Main text

### Beginnings

Natural hazard impacts have surged in recent decades, with 2023 alone seeing $250 billion in losses and over 74,000 deaths.^1^^,^^2^^,^^3^^,^^4^ Multi-(hazard-)risks, involving the interaction of multiple hazards, present complex challenges requiring a holistic, interdisciplinary approach.^5^^,^^6^ Earth Observation (EO), especially satellite imagery from missions like Copernicus Sentinel, is crucial for understanding these risks.^7^^,^^8^ The 2023 Türkiye earthquake highlighted the severe impacts of multi-hazards, exacerbated by existing vulnerabilities.^9^^,^^10^ Understanding the role of EO is vital for improving multi-(hazard-)risk assessment and enhancing community resilience.

### Motivation

#### About the EO4Multihazards project

The EO4Multihazards project, launched in September 2023 under the joint ESA-European Commission Earth System Science Initiative, aims to harness satellite EO technology to analyze multi-hazard events, including the Copernicus Sentinel series and ESA’s Earth Explorers. By focusing on four case studies (Dominica, Northeast Italian Alps, Veneto, and UK South region—so-called science cases) and translating scientific advancements into actionable insights, the project seeks to understand and mitigate the societal and ecological impacts of high-impact cascading and compounding multi-hazard events. In the project, we address three central research questions—in the further section, we summarize the input derived from the workshop and interactive poster relevant to these research questions.

During the 2024 General Assembly of the European Geophysical Union, where thousands of earth scientists, practitioners and other stakeholders gathered, the scientific coordination team of the EO4Multihazards project took advantage of the opportunity to organize a workshop to introduce the project and delve into its three primary research questions. The workshop comprised a project overview, a panel discussion, interactive group engagements, and a feedback session. Moreover, we showcased a poster with interactive components to get input from a broader audience. The audience consisted of 30 invited participants from a wide range of organizations such as NGO’s, research institutes, universities, the European Commission, and the European Space Agency. These collaborative efforts not only enriched our conversations but also broadened our understanding of EO’s pivotal role in multi-(hazard-) risk assessment and management beyond the borders of our project consortium. This document aims to summarize the essence of these discussions.

### Challenges and opportunities

#### Research question 1: What role do EO technologies, methods, data, and tools play in advancing our understanding of multi-(hazard-) risk scenarios?

The workshop underscored the pivotal role of EO technologies, methods, data, and tools in advancing our understanding of multi-(hazard-) risk scenarios. Discussions highlighted a significant shift away from a single-hazard approach toward understanding impact chains, emphasizing the importance of comprehensively assessing interconnected risks for effective mitigation strategies. EO enables precise mapping of hazards, providing empirical evidence and validation, even in data-poor regions (e.g., Woldai 2020). It facilitates comprehensive assessments by utilizing both public and private satellite data, scaling up hazard analysis, and extracting insights from past trends observable from EO. Furthermore, EO enables attribution of damage to specific hazards, analysis of exposure changes over time, and assessment of post-disaster recovery trajectories. Despite challenges such as local regulations impacting drone usage, EO technologies remain indispensable in informing resilient strategies and interventions for mitigating multi-(hazard-) risks. Integrating social-related indicators and transitioning physical data into social proxies deepens our comprehension of vulnerability dynamics and societal impacts. The workshop also emphasized the importance of effectively communicating hazard information and addressing misuse and uncertainty concerns.

#### Research question 2: What specific EO products are currently absent or needed to enhance our understanding of hot, dry, and wet multi-(hazard-) risks across diverse spatial and temporal scales?

Discussions around research question 2 underscored the urgent need for expansive spatial coverage and high-resolution data, especially in challenging terrains like mountainous regions, to understand consecutive hazard events comprehensively. Challenges include integrating diverse datasets effectively, ensuring data quality assessment, and addressing data gaps in specific environmental contexts. Participants highlighted the absence of standardized reference data to assess the quality and uncertainty of EO products, stressing the importance of having consistent methods for evaluating data quality. Moreover, the need for high spatial and temporal resolution data, particularly for multi-hazard monitoring like landslide cases, was emphasized. Lidar-based digital elevation model (DEM) data with high resolution was also appreciated for applications such as landslide monitoring and surface subsidence estimation. Another missing aspect identified was the lack of atmospheric vertical profiling information, crucial for understanding hydro-climatic extremes like storms. Additionally, discussions highlighted the importance of clarifying resolution requirements based on specific applications and scenarios, as well as the need for public entities to provide support to identify the main research gaps, especially in ensuring the availability of high-resolution datasets to complement commercially available ones. Notably, one of the central points underscored was the necessity of exploring how EO can effectively contribute to assessing socioeconomic processes and enhancing our understanding of vulnerability.

#### Research question 3: What methods are necessary to integrate EO products with (*in situ*) data and advance our understanding of multi-(hazard-) risk?

Research question 3 delves into the practical aspects of integrating EO data into hazard management workflows, emphasizing the need for standardized portals, data validation, and user-focused approaches to ensure equity in risk assessment and address unobservable risk drivers. Discussions underscored challenges in data acquisition, labeling datasets, and incorporating citizen science for anticipatory actions, highlighting the importance of harnessing EO data for vulnerability assessments through innovative methodologies. Key points included the importance of open data portals with user-friendly interfaces, the necessity of addressing biases in AI algorithms, and the value of combining EO data with *in situ* observations to tailor methods for varied risk management phases. Additionally, the conversation highlighted the significance of interpretable machine learning methodologies, the need for platforms to facilitate data sharing and processing, and the complexities of integrating technical innovations into operational workflows. Case-driven participatory approaches were proposed, alongside the recognition of the diverse needs of end users, such as first responders and decision-makers in disaster risk management, emphasizing the need for tailored methods to comprehensively assess multi-(hazard-) risks. Challenges in understanding building types from EO data were also acknowledged, emphasizing the complexity of extracting detailed structural information remotely.

### Where do we go from here?

#### Key takeaways

(1) EO’s key role: EO plays a pivotal role in understanding multi-(hazard-) risks, enabling precise mapping and comprehensive assessments.
(2) Data enhancement needs: urgent calls were made for expanded spatial coverage, high-resolution data, and standardized reference data to improve EO product quality assessment.
(3) Integration challenges: integrating EO with *in situ* data require standardized portals, machine learning, participatory approaches, and a keen understanding of user needs, despite challenges.
(4) EO for vulnerability: urgent need to explore the potential of EO to assess and monitor socioeconomic processes and components of vulnerability.

The workshop facilitated interactive discussions and engaged a diverse range of stakeholders from various backgrounds and career levels. We highly appreciate their participation and extend our gratitude to both the participants and the panel for their valuable insights.

**Figure:**
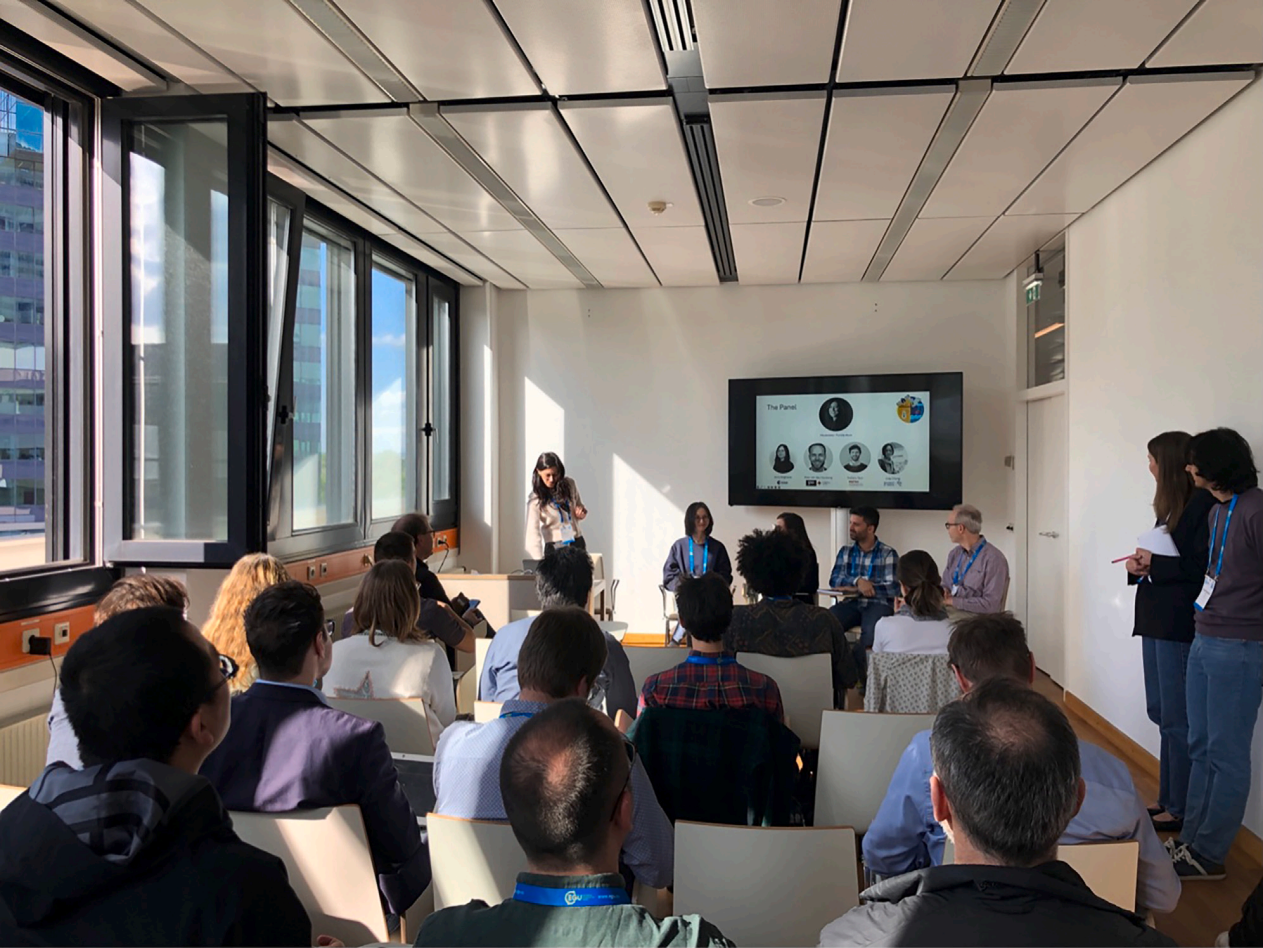
Panel discussion of the EO4Multihazards workshop moderated by Prof. Funda Atun, Dr. Ling Chang, Anca Anghelea, Dr. Stefano Terzi and Prof. Marc van den Homberg (left to right).

## Acknowledgments

We acknowledge support from the EO4Multihazards project (Earth Observation for high-impact multi-hazards science), funded by the European Space Agency and launched as part of the joint ESA-European Commission Earth System Science Initiative.
